# The effect on morbidity of the use of prophylactic abdominal drain following elective laparoscopic cholecystectomy

**DOI:** 10.12669/pjms.35.5.291

**Published:** 2019

**Authors:** Mustafa Taner Bostanci, Mehmet Saydam, Koray Kosmaz, Baki Tastan, Erdal Birol Bostanci, Musa Akoglu

**Affiliations:** 1 Mustafa Taner Bostanci, Department of General Surgery, Diskapi Yildirim Beyazıt Training and Research Hospital, Ankara, Turkey; 2 Mehmet Saydam, Department of General Surgery, Diskapi Yildirim Beyazıt Training and Research Hospital, Ankara, Turkey; 3 Koray Kosmaz, Department of General Surgery, Ankara Training and Research Hospital, Ankara, Turkey; 4 Baki Tastan, Department of General Surgery, Kayseri Training and Research Hospital, Kayseri, Turkey; 5 Erdal Birol Bostanci, Department of Gastroenterological Surgery, Turkiye Yuksek Ihtisas Training and Research Hospital, Ankara, Turkey; 6 Musa Akoglu, Department of Gastroenterological Surgery, Turkiye Yuksek Ihtisas Training and Research Hospital, Ankara, Turkey

**Keywords:** Abdominal drain, Cholecystectomy, Laparoscopy

## Abstract

**Background and Objective:**

To evaluate the clinical role of the routine use of a drain in an elective laparoscopic cholecystectomy operation applied to patients with symptomatic cholelithiasis not showing acute inflammation.

**Method:**

Following laparoscopic removal of the gallbladder, patients were separated into two groups of 30 each, either with subhepatic drain placement or without. The presence of subhepatic fluid collection was evaluated with transabdominal ultrasonography (USG) at 24 hours postoperatively and on the 7th day. The other parameters evaluated were postoperative morbidity, shoulder and abdominal pain.

**Results:**

No statistically significant difference was found between the two groups in respect of demographic characteristics and operative details. The median pain score was determined to be statistically significantly higher in the group with a drain applied compared to the group without a drain (p=0.007). In the comparison between the groups of fluid collection on USG at 24 hours and shoulder pain persisting until the 7th day, although seen less in the group with no drain applied, no statistically significant difference was determined (p=0.065, p=0.159). In the examinations made on the 7th day, no hematoma or significant fluid collection was determined on USG and no wound infection was observed in any patient of either group.

**Conclusion:**

The routine application of prophylactic subhepatic drain in laparoscopic cholecystectomy procedure did not show any benefit to the patient.

## INTRODUCTION

Currently, laparoscopic cholecystectomy is the gold standard surgical procedure for symptomatic cholelithiasis. The role of the routine use of an abdominal drain following laparoscopic cholecystectomy in reducing postoperative morbidity remains a matter of controversy. The basic reasons for routine use of an abdominal drain following laparoscopic cholecystectomy are to prevent intraperitoneal fluid collection and for the early determination of complications such as postoperative bleeding and bile leakage.^1^ Conventional cholecystectomy has also been included in the debates on this subject and several randomised studies have shown that there is no benefit to the routine use of a drain in conventional cholecystectomy.^1^

Some surgeons have considered that these outcomes obtained for conventional cholecystectomy could also be valid for a laparoscopic approach and similar results have been obtained in studies on this subject.^2^ However, there are also studies that have reported that the placement of an abdominal drain after laparoscopic cholecystectomy has ameliorated postoperative pain, nausea and vomiting by withdrawing residual intra-abdominal gas.^3^ The aim of the current study was to evaluate the effect on postoperative morbidity of the use of a drain following laparoscopic cholecystectomy in patients with cholelithiasis without acute inflammation.

## METHODS

In this prospective randomised study, a total of 60 patients who underwent laparoscopic cholecystectomy operation and met the study criteria were randomly allocated to two groups with or without drain. The allocation was made by drawing lots from a pre-prepared box of 30 drain (+) and 30 drain (-) tickets. The laparoscopic cholecystectomy procedure was applied to all patients by a single surgeon (MA) using 4 trochars and with the patient in the French position. Approval for the study was granted by the Ethics Committee of Turkey Yuksek Ihtisas Hospital (decision no 06413 dated 30.06.2014). Inclusion criteria for the study were; elective uncomplicated cholelithiasis (Grade 1, 2, 3 gallbladder), American Society of Anesthesiologists (ASA) score 1-2-3 patients, and age <70 years. Uncomplicated cholelithiasis criteria were defined by the surgeon during laparoscopic cholecystectomy according to the gall bladder adhesion scoring scale defined by Akoglu et al.^4^ Using this scale of grade 1 = no pericholecystic adhesions, grade 2 = adhesions easily loosened with dissection, grade 3= chronic pericholecystic adhesions showing fibrotic properties permitting dissection, grade 4 = adhesions preventing the easy determination of anatomic structures and making dissection difficult, which are intense accompanied by a thickened gall bladder wall (sclera atrophic cholelithiasis). Exclusion criteria were; grade 4 gallbladder with stones^4^, conversion cholecystectomy, emergency cholecystectomy, previous upper abdominal surgery, predisposition for bleeding and chronic liver disease, gangrenous and emphysematous cholecystitis, intraoperative injury or bleeding, choledocholithiasis, cholangitis, pancreatitis, and unwillingness to participate in the study.

In the study, a drain was placed intraoperatively in 30 patients and not placed in the other 30 patients. All the drains were the closed vacuum type (Jackson-Pratt©) and were applied from a five mm lateral port. After the cholecystectomy, the gall bladder was removed from a 10 mm epigastric port. Prophylaxis of cefazolin 1 gr flacon 1 x 1 and analgesia were administered to all patients.

The clinical data were recorded for all patients preoperatively in terms of age, gender, body mass index (BMI), ASA score, and operating time. Evaluation of pain was made every two hours during the postoperative 24 hours via Visual Analog Score (VAS). USG examination in respect of subhepatic hematoma and/or fluid accumulation was applied to all patients before discharge at the 24th hour and on the 7th day. From the 6 th hour postoperatively, oral intake was permitted to all patients, and the drains were removed after 24 hours (unless there was bile in the drain fluid or >100ml serous drainage or hemorrhage) and morbidity results were recorded. All patients were questioned one week after discharge in respect of persistent postoperative shoulder pain and the results were recorded.

### Statistical Analysis

Data analysis was performed using IBM SPSS Statistics version 17.0 software (IBM Corporation, Armonk, NY, USA). Conformity of continuous variables to normal distribution was determined using the Kolmogorov Smirnov test. While categorical data were shown as number of cases and percentages, descriptive statistics for continuous variables were expressed as mean ± SD or median (min-max), where applicable. The mean differences between groups were compared with the Student’s t test, and the Mann Whitney U test was applied for comparisons of variables not with normal distribution. Categorical data were analyzed with the Continuity Corrected Chi-square test or Fisher’s Exact test, as appropriate. A value of p< 0.05 was considered statistically significant.

## RESULTS

**Fig.1:**
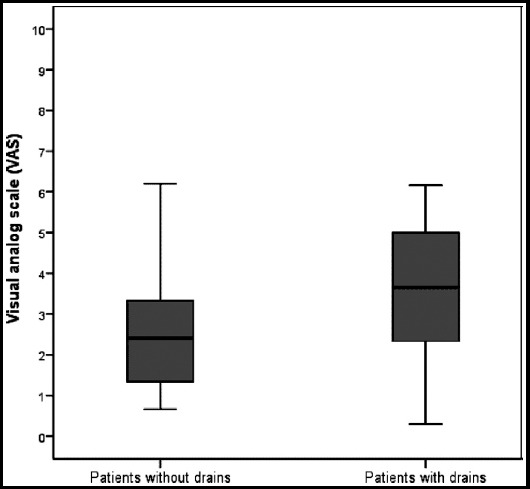
VAS scores of the groups.

No conditions were determined which would prevent drain removal, so all drains were removed at postoperative 24 hours. No statistically significant difference was determined between the groups with or without drain in respect of age (p=0.503), mean BMI (p=0.117), mean operating time (p=0.444) (Table-I).The distribution of ASA grade and gallbladder adhesion grade between the two groups was found to be statistically similar (p>0.05). The median VAS scores in the group with drain applied were determined to be statistically significantly higher than those of the patients in the group without drain (p=0.007) (Fig.1). The collection on USG at the 24th hour and the 7th day persistent shoulder pain values were seen at a lower rate in the group with drain compared to the group without drain but no statistical significance was determined (p=0.065, p=0.159) (Table-I). At the postoperative 7-day follow-up examination, no significant collection or hematoma were determined on USG and no wound infection was observed in any patient included in the study.

**Table I:** Demographic and clinical characteristics.

	Patients without drains	Patients with drains	p-value
Age (years)	46.4±12.5	48.5±12.4	0.503†
***Gender***			0.127‡
Male	10 (33.3%)	4 (13.3%)	
Female	20 (66.7%)	26 (86.7%)	
BMI (kg/m^2^)	27.7±5.4	29.5±3.6	0.117†
***ASA***			
I	19 (63.3%)	19 (63.3%)	-
II	10 (33.3%)	10 (33.3%)	-
III	1 (3.3%)	1 (3.3%)	-
***Grade***			
I	17 (56.7%)	22 (73.3%)	0.279‡
II	11 (36.7%)	6 (20.0%)	0.252‡
III	2 (6.7%)	2 (6.7%)	1.000¶
Duration of operation (min)	35.0 (15.0-75.0)	37.5 (15.0-75.0)	0.444$
VAS scores	2.41 (0.66-6.20)	3.66 (0.30-6.16)	0.007$
Shoulder pain	12 (40.0%)	6 (20.0%)	0.159‡
Post-op collection at 24^th^ hour	16 (53.3%)	8 (26.7%)	0.065‡

BMI: Body mass index, † Student’s t test, ‡ Continuity corrected Chi-square test, ¶ Fisher’s exact test, $ Mann Whitney U test.

## DISCUSSION

Cholecystectomy is one of the most frequently applied gastrointestinal operations. However, there are still limited data on the subject of the prophylactic use of a drain for laparoscopic cholecystectomy. From the past to the present-day, surgical drains have been widely used for therapeutic purposes to prevent the development or progression of intra-abdominal sepsis by removing infected debris, blood or bile after surgery for intra-abdominal infection tables such as visceral organ perforation, anastomosis leakage or liver abscess.^5^ A peritoneal drain was applied as a part of the operation in cholecystectomy surgery first performed by Lamgenebuch in 1882. Routine drain placement in conventional cholecystectomy has been accepted and applied as part of the procedure for many years.^6^ Conventional cholecystectomy without the application of subhepatic drainage was first described in 1913 and from that date onwards for many years there was no consensus on the necessity to apply drainage routinely or only in complicated cases in conventional cholecystectomy. However, in several subsequent, randomised, controlled studies, the application of routine subhepatic drainage was shown to have no benefit in conventional cholecystectomy.^6^

After the introduction of laparoscopic cholecystectomy at the beginning of the 1990s, the laparoscopic technique replaced the conventional technique and became the gold standard technique for symptomatic cholelithiasis. However, the majority of surgeons continued to routinely apply subhepatic drainage after laparoscopic cholecystectomy on the basis of their experience and old beliefs. A drain is traditionally used after cholecystectomy for the early detection of bile leakage and unexpected bleeding and to drain abdominal fluid collection. One of the main reasons for drain use is the concern of potential bile leakage that could lead to biliary peritonitis. Bile leakage is generally because of an aberrant bile canal or a cystic incomplete canal. Currently, biliary complications following laparoscopic cholecystectomy are at the level of 0.4% (0.1%-0.9%) and therefore, the clinical efficacy of drain use is considered to be minimal.^7^ Furthermore, extensive clinical series studies of conventional cholecystectomy have shown that because of post-cholecystectomy biliary peritonitis, the placement of a drain in the majority of patients where laparotomy has been made, has not been a significant determinant in the prevention of this complication.^8^ It is assumed that the use of a drain could be useful in the early determination of postoperative bleeding. Although this has implications for patients with evident bleeding, there can be early determination of intra-abdominal bleeding in patients without a drain both clinically and sonographically.^9^

Another principal reason for the use of a drain is to prevent the development of intra-abdominal collection following laparoscopic cholecystectomy. Generally, the peritoneal cavity rapidly absorbs serous fluids. Post-cholecystectomy collections in the subhepatic area are often small and can be rapidly absorbed. It has been shown that many post-cholecystectomy collections are asymptomatic and are absorbed by the peritoneum.^9^ However, experimental studies have shown that drains placed in the peritoneal cavity when there is no fluid accumulation become rapidly encircled by the omentum and become occluded in a short time.^10^ The use of a drain following laparoscopic cholecystectomy may have increased the development of collection in the subhepatic area in a study by Georgiou et al.^11^ Possible reasons for this were considered to be irritation which developed related to the foreign body property of the drain, the creation of a dead cavity and the absorption effect of the drain.

There are studies that have stated that the drain could even be one of the potential causes of bile leakage.^12^ In the current study, although subhepatic collection/hematoma was seen at a lower rate at the postoperative 24 th hour in patients with a drain, the difference between the two groups was not statistically significant and on the USG examination on the 7th day postoperatively, no subhepatic collection or hematoma were determined in any patient of either group. Another point of debate is the effect on postoperative nausea and vomiting and pain of the application of a subhepatic drain after laparoscopic cholecystectomy. Postoperative pain and postoperative nausea and vomiting are significant problems causing discomfort and as such are the most significant causes of delayed discharge following laparoscopic procedures. It has been suggested that pneumoperitoneum provided by CO2 insufflation is one of the causes of nausea, vomiting and postoperative pain, primarily shoulder pain.^13^,^14^ Many surgeons believe that by reducing the amount of residual gas, closed vacuum drains are useful in decreasing postoperative nausea and vomiting and postoperative shoulder pain.^14^ However, in some randomised, controlled studies, no relationship has been found between the drain and a reduction in postoperative nausea, vomiting and shoulder pain.^15^,^16^ In a study by Barczynski et al, postoperative pain was determined to be immediately reduced on removal of the drain and this was reported to be related to the mechanical irritation of the drain.^17^ In the current study, the postoperative first 24-hour VAS scores of the patients in the group with drain applied were observed to be higher. The higher pain level in this group can be considered to be due to the mechanical irritation of the drain. Furthermore, although not statistically significant, that less shoulder pain was seen in the group with the drain showed that the drain played a partial role in removing subdiaphragmatic residual gas.


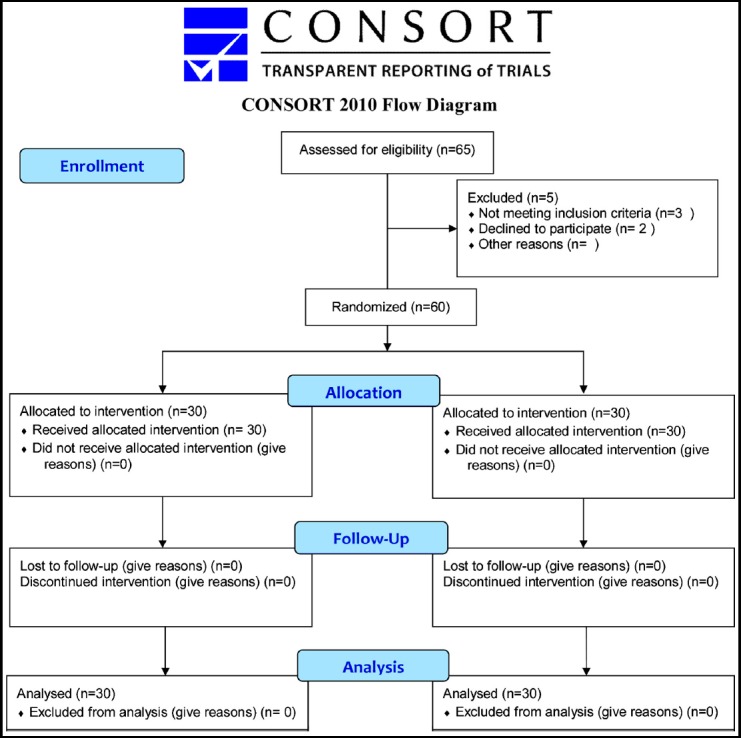


Port site infection has been reported to be seen in 1.1%- 7.9% of patients following laparoscopic cholecystectomy.^18^ Several factors may play a role in the development of port site infection, such as cholecystitis, removal of the gall bladder from the abdomen with the aid of an endobag, gall bladder perforation and foreign body reaction caused by the drain. Some studies have reported that drain use increases infectious complications. This is thought to be due to the drain having become a bacterial entry site to the abdominal cavity over time.^18^ However, studies have shown that wound site infection has not developed with short-term drain usage after either conventional or laparoscopic cholecystectomies.^19^ Otherwise, Bugiantella et al. showed that the subhepatic drainage after elective uncomplicated laparoscopic cholecystectomy had no affect on wound site infection.^20^ In the current study, no wound site infection developed in any patient of either group.

In some previous studies, drain usage has been reported to cause patient discomfort and extend the length of stay in hospital.^20^-^22^ All the patients in the current study were discharged the following day and there were no readmissions in the early postoperative period. However, patient comfort was observed to be worse in the group where the drain was applied.

Ishikawa et al,^2^ reported drain usage after laparoscopic cholecystectomy in cases defined as complicated, such as intraoperative excessive bleeding, difficult operations, and intraoperative spread of bile.^4^ According to the experience and data obtained from this study, drain usage is indicated in cases such as acute complicated cholecystitis, elective scleroatrophic cholelithiasis, when smooth closure cannot be made of a cystic canal, when there is perioperative injury or bleeding, or a history of anticoagulant use. According to the findings of this study, it can be said that drain usage in cases of elective non-complicated cholelithiasis does not provide any additional benefit to the patient. Patient comfort is impaired by abdominal pain associated with irritation. Moreover, concerns that have directed surgeons to prophylactic drain use can now be determined clinically and radiologically and eliminated with non-operative methods.

## CONCLUSION

In conclusion, when the data of this randomised controlled study are evaluated together with the information in literature, no benefit for the patient was found from the routine use of prophylactic subhepatic drain in a laparoscopic cholecystectomy procedure. As there is no assurance on the subject of prevention and treatment of postoperative collection, bleeding and biliary peritonitis in elective, non-complicated cases of cholelithiasis, drainage should be used selectively.

### Author`s Contribution

**MS and MTB** conceived, designed and did statistical analysis & editing of manuscript.

**KK and BT** did data collection and manuscript writing.

**EBB and MA** did review and final approval of manuscript.
